# Nuclear enlargement after confined migration in cancer cells

**DOI:** 10.17912/micropub.biology.001623

**Published:** 2025-11-17

**Authors:** Akane Iizuka, Naotaka Nakazawa

**Affiliations:** 1 Graduate School of Science and Engineering, Kindai University, HigashiOsaka-city, Osaka, Japan; 2 Faculty of Science and Engineering, Kindai University, Higashiosaka-city, Osaka, Japan

## Abstract

Aggressive cell migration is a hallmark of cancer cells. Cancer cells pass through a 3D confined environment during metastasis, which induces mechanical stress on the nucleus. Transwell membranes have been used in research to induce mechanical stress by cell migration in 3D confined space, but other products with microscale pores have not been widely investigated. Here, we report how cancer cells respond to mechanical stress using a TC insert with microscale pores that mimic a 3D confined extracellular environment. As shown in previous studies using the Transwell membranes, our results indicate that the size of microscale pores in the TC insert modulates cancer cell penetration rates and nuclear morphology. Thus, the TC insert is also a useful option to induce mechanical stress by cell migration in a 3D confined environment.

**Figure 1. Confined migration of U2OS cells in a TC insert with different micron pores:**
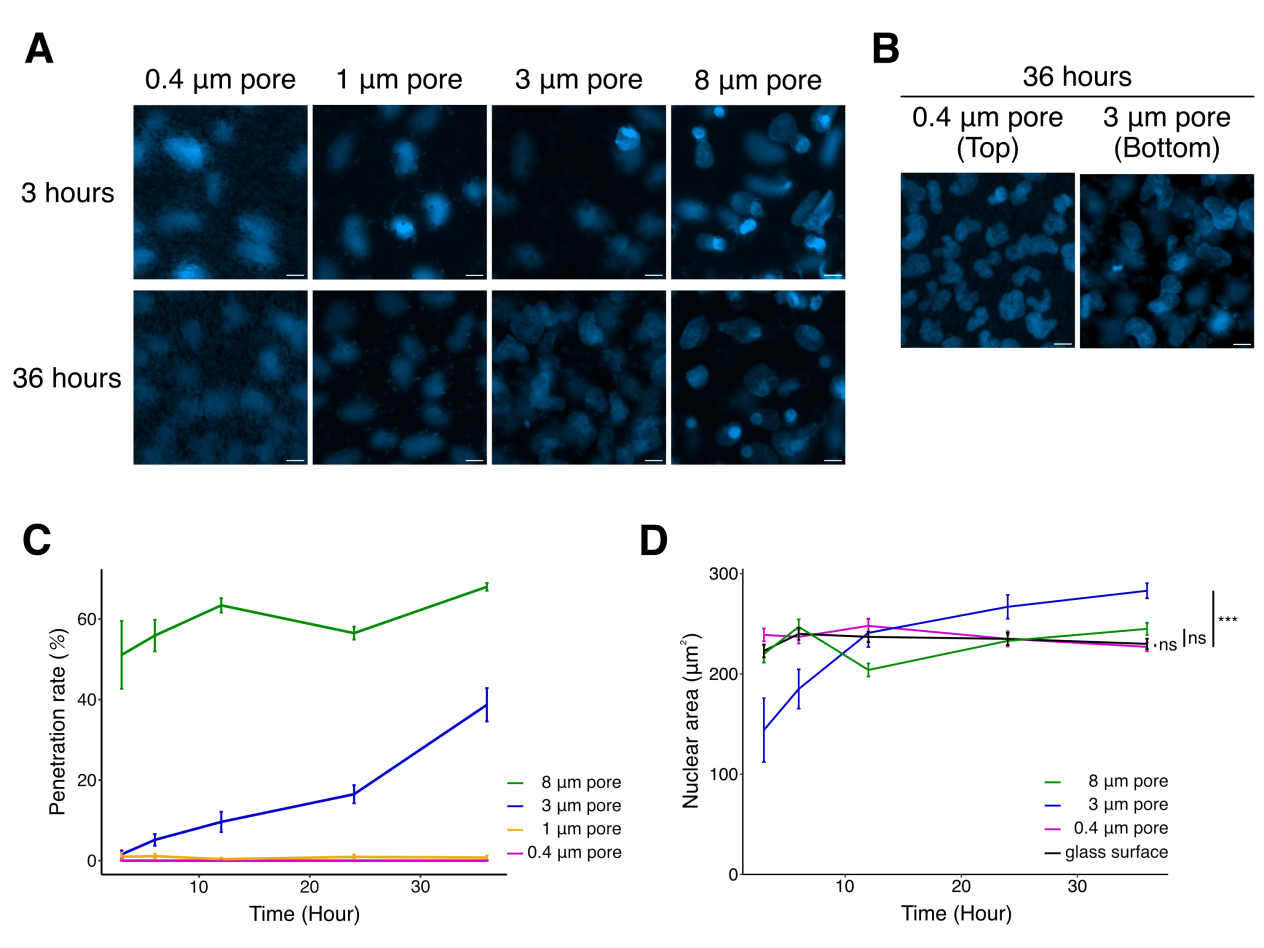
(A)&nbsp; Fluorescent images of the nucleus (Blue) of U2OS cells with focusing on the bottom of the TC insert membranes 3 hours or 36 hours after cell seeding. The scale bars are 10 μm. (B)&nbsp; Fluorescent images of the nucleus (Blue) of U2OS cells with focusing on the nucleus on the top (0.4 μm pores) or the bottom (3 μm pores) of the TC insert membranes 36 hours after cell seeding. The scale bars are 10 μm. (C)&nbsp; A graph showing the relationship between the penetration rate of U2OS cells through the TC insert membrane with pores (Magenta: 0.4 μm, Yellow: 1 μm, Blue: 3 μm, and Green: 8 μm) and at different time intervals (3, 6, 12, 24, 36 hours). Over 60 cells were counted. Samples were collected from three independent experiments. (D)&nbsp; A graph showing the cross-sectional area of the cell nuclei that passed through the TC insert membrane in the same samples as in (A) and glass surfaces (Black: Glass surface, Magenta: 0.4 μm, Blue: 3 μm, and Green: 8 μm). The data with 0.4 μm pores represents the cross-sectional area of cells on the top of the membrane, while the data with 3 μm and 8 μm pores sizes represent the cross-sectional area of cell nuclei at the bottom of the membrane. Over 6 cells were counted. Samples were collected from three independent experiments. ***<0.001, Dunnett’s test.

## Description

Cell migration is a critical cellular function that is involved in several physiological processes. Migrating cells sometimes pass through a space that is much smaller than their own, accompanied by a large deformation in the shape of the cell. As a result, the nucleus, the largest organelle in the cell, is under mechanical stress, leading to nuclear deformation (Yamada and Sixt, 2019). Importantly, nuclear deformation triggers a sensing mechanism of the 3D cell space through an increase in intracellular calcium, cPLA2 activation, and production of arachidonic acid, resulting in increased actomyosin contraction for cell migration (Lomakin et al., 2020; Venturini et al., 2020). Additionally, the 3D confinement of the nucleus induces mechanical stress, which leads to DNA damage and repair, accompanied by nuclear membrane rupture (Denais et al., 2016; Raab et al., 2016). This leads to the mislocalization of DNA repair factors and genomic heterogeneity (Irianto et al., 2017). Thus, mechanical impacts on cancer cells in the microenvironment are thought to be important for new insights into understanding cancer progression and treatment (Linke et al., 2024).

Microporous membranes such as polyester (PET), polycarbonate (PC), and polytetrafluoroethylene (PTFE) with micron-sized pores have been used for *in vitro* cell migration and invasion assays (Pijuan et al., 2019; Dogan and Dufva, 2022). The Transwell permeable supports from Corning have been the most widely used for inducing mechanical stress in various types of migratory cell, including primary cultured neurons (Lomakin et al., 2020; Kamikawa et al., 2023; Kengaku et al., 2025; Nakazawa et al., 2025; Zhang et al., 2025). However, other commercially available microporous membranes with small pores have not been widely examined.

Here, we report the effects of TC inserts manufactured by SARSTEDT on U2OS cells, a human osteosarcoma cell line. In this study, U2OS cells were cultured on the the TC inserts with different pore sizes (0.4 μm, 1 μm, 3 μm, and 8 μm pores) for 3 to 36 hours ( Figure 1A ). To quantitatively compare transmigration efficiency, the penetration rate was examined at each condition. This rate is calculated by dividing the number of cells in the bottom compartment divided by the total number of cells in the top and bottom compartments of the TC inserts, as previously used to analyze the Transwell membranes (Nakazawa et al., 2025). As shown in Figure 1A, U2OS cells exhibited an inability to pass through the pores in the case of 0.4 μm and 1 μm pores. Following a seeding period of 3 hours and 36 hours, the majority of cells were observed to be situated on the top surface of the membrane ( Figure 1A ). Our quantitative analysis show that U2OS cells are rarely able to pass through 0.4 μm and 1 μm pores (0.4 μm; 0.00±0.00% at 3 hours, 0.00±0.00% at 6 hours, 0.00±0.00% at 12 hours, 0.00±0.00% at 24 hours, 0.00±0.00% at 36 hours, 1 μm; 0.980±0.980% at 3 hours, 1.12±0.564% at 6 hours, 0.373±0.373% at 12 hours, 0.935±0.549% at 24 hours, 0.766±0.541% at 36 hours). In contrast, the penetration rate in 3 μm increased with time (3 μm; 1.57±0.986% at 3 hours, 5.16±1.46% at 6 hours, 9.63±2.52% at 12 hours, 16.5±2.25% at 24 hours, 38.7±4.14% at 36 hours). In the case of 8 μm pores, majority of seeded cell passed through in 3 hours, and the penetration rate did not increase as much over time (8 μm; 51.1±8.42% at 3 hours, 55.9±3.93% at 6 hours, 63.4±1.80% at 12 hours, 56.5±1.61% at 24 hours, 68.0±0.956% at 36 hours). These results suggest that a comparison between 3 μm and 8 μm pores are suitable for assessing the mechanical effects on U2OS cells due to the difference in spatial size. This result is representative of the migration assay using the Transwell membrane, as shown in previous studies (Irianto et al., 2017). Taken together, TC inserts are also useful for the confined migration assay.

To further investigate the mechanical impacts of confined migration using the TC inserts, we quantified the cross-sectional area of the nucleus in U2OS cells. First, we checked if cell culture on the TC insert affects to nuclear size of U2OS cells. Our quantitative analysis indicates that the cross-sectional area of the nuclei in U2OS cells on a membrane with 0.4 µm pores was equivalent to that of cells cultured on a glass surface at each designated time point (0.4 µm; 239 ± 6.44 µm ^2^ at 3 hours, 237 ± 6.73 µm ^2^ at 6 hours, 248 ± 6.47 µm ^2^ at 12 hours, 235± 7.57 μm ^2^ at 24 hours and 227± 4.46 μm ^2^ at 36 hours, Glass surface; 223 ± 6.46 µm ^2^ at 3 hours, 240 ± 6.11 µm ^2^ at 6 hours, 237 ± 5.17 µm ^2^ at 12 hours, 235 ± 6.13 µm ^2^ at 24 hours, 230 ± 5.26 µm ^2^ at 36 hours). A comparison of the cross-sectional area of the nuclei under the condition between glass surface and the top of the membrane with 0.4 µm pores at 36 hours reveals no significant differences ( Figure 1D ). These results suggest that culturing cells on the TC insert membrane does not affect the baseline of nuclear size. Consequently, the cross-sectional area of the nuclei in cells that did not pass through the 0.4 µm pores was utilized, as a control because the observation on the top surface with 0.4 µm pores appear to be analogous to 2D conditions. In order to facilitate a comparison between the culture conditions with 0.4 µm pores and 8 µm pores, the cross-sectional area of the nuclei in cells that passed through the 8 µm pores was examined over time. The results demonstrated that, over time, the cross-sectional area of the nuclei in cells that passed through the 8 µm pores became similar to that of cells that did not pass through the 0.4 µm pores (8 µm; 220 ± 8.67 µm ^2^ at 3 hours, 247 ± 7.55 µm ^2^ at 6 hours, 204 ± 6.49 µm ^2^ at 12 hours, 233 ± 6.16 µm ^2^ at 24 hours, 245 ± 6.12 µm ^2^ at 36 hours) ( Figure 1D ). In contrast, the nuclear area of cells passing through 3 µm was small at 6 hours after cell seeding, but it became closer to that of cells on the membrane with 0.4 μm pores over time at 12 hours (3 μm; 144±31.9μm ^2^ at 3 hours, 185±19.6μm ^2^ at 6 hours, 241±14.1μm ^2^ at 12 hours) ( Figure 1D ). In addition, the nuclear area of cells that passing through 3 µm pores became significantly larger than that of cells at the top of the 0.4 µm membrane after 36 hours of cell seeding (3 μm; 267±11.9 μm ^2^ at 24 hours, 283±7.50 μm ^2^ at 36 hours) ( Figure 1B and 1D). A previous study suggested that the nuclear area after migration in 3 µm pores in the Transwell membrane was restored after 7 days of cell seeding (Irianto et al., 2017). Combined with our results and a previous study, the nuclear size may fluctuate before restoration to the original nuclear size. Since confined migration affects the cell cycle, the fluctuation of nuclear area may be related to the stage of the cell cycle (Xia et al., 2019; Bastianello et al., 2024).

In this study, we investigated the penetration rate of U2OS cells in confined space using the TC inserts and the effects of confined migration on the nuclear size. Our results show that migration assay using the TC inserts with 3 μm and 8 μm pores is also available for confined migration assay as well as using the Transwell membrane. In the future, the TC insert is also an option for the experiments to evaluate the change of chromatin state and gene expression by mechanical stress in confined migration (Irianto et al., 2017; Fanfone et al., 2022).

## Methods

**ECM coating of TC inserts:** Polystyrene 24-well cell culture plates (VIOLAMO) and polyester TC inserts (SARSTEDT) with four different pore sizes (0.4 µm, 1 µm, 3 µm, 8 µm) were employed. Ahead of fibronectin coating, the TC inserts were coated with 100 µL of Poly-D-Lysine (PDL) (Sigma-Aldrich, p6407) on the top of the membrane and 600 µL of PDL solution on the bottom of the membrane for 12 hours. Cover glasses (12 mm Round, Matsunami) were coated with 500 µL of Poly-D-Lysine (PDL) for 12 hours. After PDL coating, the inserts or cover glasses were coated with Fibronectin (Corning, 354008) for 12 hours. Finally, the inserts were incubated with McCoy’s 5A medium for 12 hours.

&nbsp;

**Cell culture:** U2OS cells, differentiated osteosarcoma cell line, were obtained from ECACC (EC92022711-F0). U2OS cells (2.9 × 10 ^4^ cells per 1 well) were seeded in 100 µL of medium on the TC inserts and cultured for 3 hours, 6 hours, 12 hours, 24 hours, and 36 hours, respectively. At each time point, the cells were washed with phosphate-buffered saline (PBS) and fixed with 4% paraformaldehyde in PBS in both the upper and lower chambers for 30 minutes at room temperature.

^&nbsp;^

**Nuclear staining and imaging:** The cells were washed thoroughly with PBS and treated with PBS with 0.1% Triton X for 10 minutes at room temperature. The cells were then washed again with PBS and stained with DAPI solution (Dojindo, D523) in water (1/5000) for 30 minutes at room temperature. After additional washing with Milli-Q water, 20 µL of DAPI Fluoromount-GR (Southern Biotech) was applied to the cells. For imaging, the TC inserts were carefully cut with a V-Lance ^TM^ Knife (disposable ophthalmic knife) and interposed using two glass coverslips. The cells were observed using Axio Observer7 with a ×20 lens (NA: 0.50) (Zeiss).

&nbsp;

**Image analyses and statistical analyses:** Image analyses were performed using Fiji (ImageJ 2.0) or the ZEN application (Zeiss). Data were analyzed using R software.

&nbsp;

**Quantitative analyses for transmigration efficiency:** We randomly selected the imaging compartment (404 μm × 337 μm) on the TC inserts. The cell numbers on the top and bottom surfaces of the compartment were counted. We defined the penetration rate as the transmigration efficiency, which was calculated by dividing the number of cells in the bottom compartment by the total number of cells on the top and bottom compartments.

&nbsp;

**Quantitative analyses for the cross-sectional area of the nuclei:** The nuclei of U2OS cells on the top surface (0.4 μm pores), on the bottom surface (3 μm pores and 8 μm pores), and on cover glasses were randomly selected for imaging in a compartment (404 μm × 337 μm). The area of the cell nucleus center in the z-direction was quantified as the "nuclear cross-sectional area".
